# Strongyloidiasis in a Patient Diagnosed by Metagenomic Next-Generation Sequencing: A Case Report

**DOI:** 10.3389/fmed.2022.835252

**Published:** 2022-04-07

**Authors:** Junyan Qu, Zhiyong Zong

**Affiliations:** ^1^Center of Infectious Diseases, West China Hospital, Sichuan University, Chengdu, China; ^2^Center for Pathogen Research, West China Hospital, Sichuan University, Chengdu, China

**Keywords:** *Strongyloides stercoralis*, strongyloidiasis, metagenomics next generation sequencing, cerebrospinal fluid, albendazole

## Abstract

**Background:**

Strongylodiasis may be asymptomatic or cause mild gastrointestinal symptoms, and may be a fatal disseminated disease or *Strongyloides* hyperinfection syndrome. Non-specific clinical manifestations, such as pneumonia and gastroenteritis, pose a diagnostic dilemma.

**Case Presentation:**

We report a case of a 67-year-old Chinese male who presented with abdominal pain, fever, headache, vomiting, constipation, and slight cough with sputum for nearly 2 months. He had been in good health and had no history of glucocorticoid use. He was diagnosed with enterococcal meningitis and intestinal obstruction at a local hospital and improved after treatment with vancomycin, but symptoms of headache and abdominal pain soon recurred. The metagenomic next-generation sequencing (mNGS) of the cerebrospinal fluid using Illumina X10 sequencer revealed seven sequence reads matching *Strongyloides stercoralis.* Strongyloidiasis was suspected. Microscopic examination of gastric fluid revealed the presence of *S. stercoralis* larvae, which was confirmed by PCR to amplify both *S. stercoralis* ribosomal DNA gene and mitochondrial cytochrome c oxidase subunit 1 gene and sequencing amplicons. Strongyloidiasis was diagnosed. Albendazole (400 mg, twice daily) was used, and the patient recovered gradually.

**Conclusion:**

mNGS may be a useful tool for detecting uncommon infectious disease. The case would help clinicians to raise awareness of strongyloidiasis in non-endemic areas and reduce fatality.

## Background

*Strongyloides stercoralis* is a soil-transmitted helminth that can persist and replicate in humans and cause strongyloidiasis. It is endemic in tropical and subtropical areas including parts of China and areas with limited resources (1). Chinese cases have been reported mainly in Guangxi and Yunnan provinces in southern China (2, 3). Strongyloidiasis may be asymptomatic or cause mild gastrointestinal or pulmonary symptoms in immunocompetent hosts, while in immunocompromised hosts it may present as a fatal disseminated disease or *Strongyloides* hyperinfection syndrome (SHS) (4, 5). The mortality rate for SHS was approximately 60% and can be up to 100% if untreated (6). The lack of disease-specific manifestations makes the diagnosis of strongyloidiasis challenging, especially in non-endemic areas.

The common diagnostic methods of strongyloidiasis include fecal examination for strongyloides larvae and serological approaches (7). However, the sensitivity of fecal examination is low (8), and feces cannot be obtained in patients with intestinal obstruction. It is also difficult for physicians in non-endemic areas to think of using serological screening methods when they are not considering strongyloidiasis. With the development of metagenomic next-generation sequencing (mNGS) technology in recent years, it has gradually become an attractive and promising approach for pathogen detection (9). mNGS of cerebrospinal fluid (CSF) has been shown to improve the diagnosis of neurologic infections (10).

Here, we describe a case of strongyloidiasis with intestinal obstruction in a non-endemic area within the country of China. He was first diagnosed by mNGS of CSF and later confirmed by gastric fluid microscopy and polymerase chain reaction (PCR) with amplicon sequencing.

## Case Presentation

**FIGURE 1:**
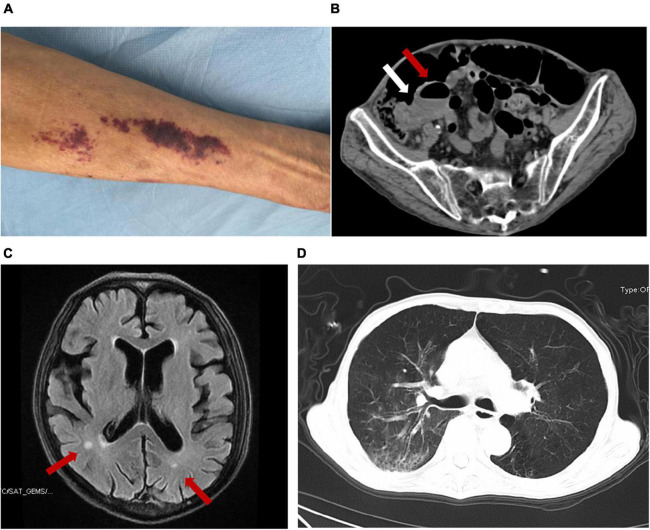
The presentation of this case and the detection of *S. stercoralis* larvae. Anaphylactoid purpura were present on left upper limb **(A)**. Abdominal CT imaging showed that the ileocecum was swollen and thickened (white arrow) and the proximal small intestine was obstructed with air-fluid level (red arrow) **(B)**. Brain MRI showed patches near bilateral ventricles (red arrow) and partial endocranium thickened **(C)**. Chest CT revealed bronchiectasis with secondary pulmonary infection **(D)**.

A 67-year-old Chinese male was admitted to our hospital for nearly 2 months of abdominal pain, fever, headache, vomiting, constipation, and slight cough with sputum but without shortness of breath. He is a farmer living on the outskirts of Chengdu, Sichuan Province, southwest China. He had no significant family history, had no underlying diseases, did not use glucocorticoids or immunosuppressive agents, did not drink alcohol, and had not traveled outside Sichuan, a non-endemic area of *S. stercoralis*. CSF analysis at a local hospital 2 months prior to the admission showed increased white blood cells (WBC, 3,534 cell/μl) and proteins (1.97 g/L) and a positive CSF culture with *Enterococcus avium.* He was diagnosed with bacterial meningitis and intestinal obstruction there. His condition improved after receiving vancomycin but 2 days prior to the admission he had recurrent, severe vomiting and abdominal pain and therefore was transferred to our hospital for referral. The patient had a 10-kg weight loss in the previous 2 months. On examination, he was afebrile but had neck stiffness. Anaphylactoid purpura were present on both upper limbs (Figure 1A) and facial skin. Signs of meningeal irritation were positive. Crackles were heard bilaterally in the lower lungs on auscultation. Right lower abdominal tenderness and scant bowel sounds were noted. His routine blood work on admission revealed 7.2 × 10^9^/L WBC (normal range, 3.5–9.5 × 10^9^/L) with 86.8% neutrophils, 7.4% lymphocytes and 0% eosinophils, and moderate anemia with 80 g/L hemoglobin. Lymphocyte test showed a decreased CD4+ T cell count of 168 cell/μl (normal range, 471–1,220 cell/μl), CD8+ T cell count of 149 cell/μl (normal range, 303–1,003 cell/μl), B cell count of 24 cell/μl (normal range, 175–332 cell/μl), and normal NK cell of 162 cell/μl (normal range, 154–768 cell/μl). Serum tests for human immunodeficiency virus (HIV), hepatitis B virus (HBV), hepatitis C virus (HCV) and syphilis were negative. PCR testing of a blood sample for human T lymphocyte virus 1 (HTLV-1) was also negative. In blood chemistry, bilirubins, alanine and aspartate aminotransferases, creatine, and urea were all in the normal range. Enhanced brain magnetic resonance imaging (MRI) showed a few patches near bilateral ventricles and partial thickening and strengthening of the endocranium (Figure 1B). An enhanced abdominal computer tomography (CT) scan exhibited proximal small intestinal obstruction (Figure 1C). A CT scan of the chest revealed bronchiectasis with secondary pulmonary infection (Figure 1D). Lumbar puncture on admission revealed clear CSF with 20 red blood cells and 18 WBC per microliter. Meningitis was diagnosed and meropenem was given at 2 g every 8 h for empirical therapy. Small bowel obstruction management measures including gastrointestinal decompression and fluid resuscitation were also given.

**FIGURE 2:**
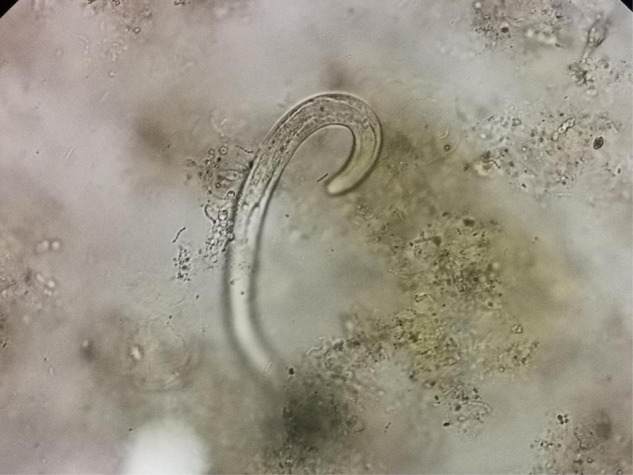
Larvae of *S. stercoralis* were identified in gastric fluid (unstained, ×400).

**FIGURE 3:**
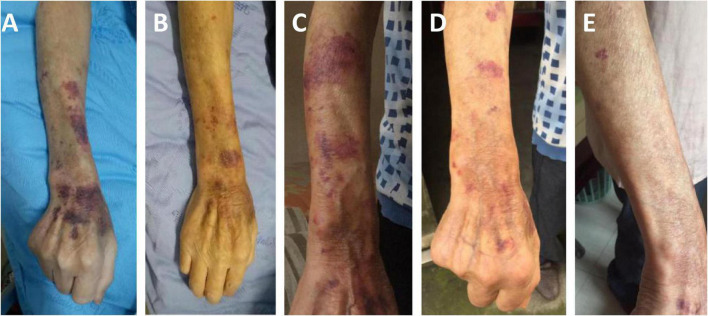
The changes of purpura in right forearm of the patient. Schonlein purpura in his right forearm at admission **(A)**, after 10 days of treatment **(B)**, 5 months after discharge **(C)**, after the second course of treatment **(D)**, one and a half months after the second course of treatment **(E)**.

**FIGURE 4:**
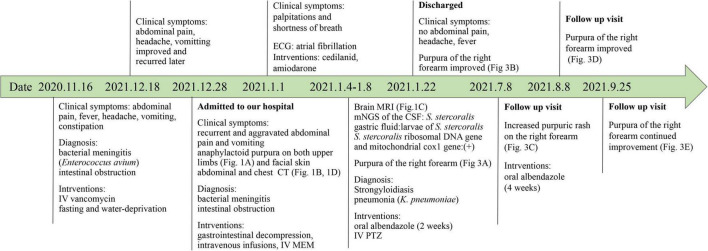
Timeline of the patient with relevant data of the past episodes and interventions. CSF, Cerebrospinal fluid; CT, computer tomography; IV, intravenous; MEM, meropenem; mNGS, metagenomic next-generation sequencing; MRI, magnetic resonance imaging; PTZ, piperacillin tazobactam.

Two days after admission, the patient developed palpitations and shortness of breath. The respiratory rate was 32 breaths per minute, the blood pressure 85/55 mmHg, heart rate 160 beats per minute, and the oxygen saturation 97% while he was breathing ambient air. His hemoglobin level was 64 g/L (reference range, 120–160 g/L). His brain natriuretic peptide and troponin-T levels increased to 6,825 ng/L (normal range, 0–227 ng/L) and 34.0 ng/L (normal range, 0–14 ng/L), respectively. Electrocardiogram indicated atrial fibrillation, which improved after administration of cedilanid and amiodarone. Three sputum smear tests were negative for bacteria, fungi, acid-fast bacilli, ova and parasites. Aerobic culture of sputum grew *Klebsiella pneumoniae*. Because the patient has a long course of disease and the etiology of intracranial infection is unknown, mNGS of the CSF was performed using an Illumina X10 sequencer with a unilateral read length of 75 bp. The results of mNGS were available on the third day after admission and among the 10,656,825 generated clean reads, there were seven sequence reads matching *Strongyloides stercoralis* but no reads matched any other parasites or microorganisms. *S. stercoralis* larvae were identified in gastric fluid retrieved from the gastrointestinal tube on the third day after admission (Figure 2). DNA extracted of larvae using a DNeasy Blood & Tissue Kit (QIAGEN, Hilden, Germany) was subjected to PCR amplification for both *S. stercoralis* ribosomal DNA gene and mitochondrial cytochrome c oxidase subunit 1 (*cox1*) gene as described previously (11, 12). Both PCR assays were positive and sequencing amplicons confirmed the presence of *S. stercoralis* with the ribosomal DNA sequence the *cox1* sequence being deposited in GenBank under accession nos. MW604696 and MW578283, respectively. Strongyloidiasis was therefore diagnosed. As ivermectin and mebendazole were unavailable for human use in China, 2 weeks of albendazole (400 mg, twice daily) was used instead. The patient recovered gradually. Five months after discharge, he gained 5 kg in outpatient follow-up, but he had new purpura on both upper limbs (Figure 3). He was given another course of albendazole for 4 weeks and gradually improved. The timeline of the patient with relevant data of the episodes and interventions is presented in Figure 4.

## Discussion

Strongyloidiasis is mainly endemic in tropical and subtropical regions and sporadic in temperate regions. In particular, a high incidence of strongyloidiasis is present in South America and Southeast Asia (13). Few cases have been reported from Sichuan provinces, southwest China. Our patient is a farmer living in Chengdu with a subtropical climate, which is not an endemic area of strongyloidiasis as only sporadic cases have been reported locally (3) and a province-wide surveillance for 3- to 6-year-old children by stool sampling revealed a 0.05% (6 out of 11,403 children) prevalence of *S. stercoralis* (14). In non-endemic areas, *S. stercoralis* infection is often neglected and not included in differential diagnoses for patients with intestinal obstruction, rashes, pneumonia and meningitis. As coincident infections with enteric organisms including *Enterococcus* spp. are common (15, 16), the symptoms of meningitis and intestinal obstruction in the patient 2 months prior to the present admission may indicate *S. stercoralis* infection. HIV or HTLV-1 infection, malignancy, organ transplant and alcoholism are common risk factors for *S. stercoralis* hyperinfection syndrome (6). In contrast, this patient has no such conditions or any other known immunocompromised factors. Although the patient had lymphocytopenia at admission, it may have been secondary to the infection, as his lymphocytes rebounded when his condition improved. Previous studies have found that eosinophilia generally occurs in asymptomatic and acute strongyloidiasis patients (17). However, patients with SHS or disseminated diseases often have no eosinophilia (18), making the diagnosis of strongyloidiasis more challenging.

Stool examination for larvae, serology and molecular biologic techniques can all be used to diagnose *S. stercoralis* infection (7, 19). As the patient had intestinal obstruction and no stools were available, we therefore used gastric fluid and CSF for examination. Although *S. stercoralis* larvae were viewed in gastric fluid but not CSF in this patient, previous studies have demonstrated low sensitivity of gastric fluid and CSF for viewing *S. stercoralis* (3, 8). Unfortunately, in non-endemic areas, serological methods are usually not available as in our hospital. As a new pathogen detection method, mNGS has become increasingly valuable in the detection of infectious diseases due to its advantages of high throughput and the ability to detect almost all kinds of microorganisms and parasites. mNGS is particularly advantageous in the detection of emerging, hard-to-culture, atypical and rare pathogens, such as *Mycobacterium tuberculosis*, non-tuberculous mycobacteria, *Mycobacterium leprae*, *Fasciola hepatica*, and *Angiostrongylus cantonensis* (20–24). However, there are no reports of strongyloidiasis diagnosed by mNGS. In recent years, mNGS has been increasingly being used in clinical practice owing to rapid technological developments and substantially reduced costs in China, therefore, mNGS may be a useful tool for detecting *S. stercoralis* in non-endemic areas.

*Strongyloides stercoralis* DNA was detected by mNGS in CSF but this may not be sufficient to diagnose *S. stercoralis* meningitis due to disseminated strongyloidiasis as larvae were no detected in CSF (8). It is possible that *S. stercoralis* larvae penetrated human intestinal wall and therefore released a small amount of cell-free DNA, which could enter the CSF when the blood-brain barrier was compromised. The patient has intestinal strongyloidiasis and bacterial meningitis. It is likely that he had developed SHS as he had concurrent cutaneous, pulmonary, and gastrointestinal symptoms (6).

Therapeutic options for strongyloidiasis are limited and typically include ivermectin and benzimidazoles (albendazole and thiabendazole). Ivermectin is currently the most effective treatment for strongyloidiasis (16, 25), but human preparations of oral ivermectin are not available in some countries including China at present, and oral veterinary preparations have been attempted in some cases (26). In addition, veterinary preparations involve some risk of overdosing, particularly when more concentrated formulations for large animal species, such as cattle, are not properly diluted. As ivermectin is not available, we administered oral albendazole, and the treatment was successful. Perhaps because of intracranial involvement, this patient was treated with albendazole for a longer duration than previously reported patients (3). Albendazole may be an alternative option for strongyloidiasis as suggested before (27, 28) but the efficacy warrants further study.

We are aware of limitations of this report. First, we did not obtain samples other than CSF and gastric fluid to examine *S. stercoralis* and we therefore did not have sufficient evidence to demonstrate the presence or absence of the in-host circulation of *S. stercoralis*. Second, the exact reasons or sources of strongyloidiasis remain unknown. Third, the CSF sample collected in the local hospital was not available for mNGS to demonstrate whether *S. stercoralis* DNA was present then though it is likely.

## Conclusion

In summary, we present the case of a patient from a non-endemic area with a delayed diagnosis of strongyloidiasis. Owing to its increased use in some countries like China, mNGS may be a useful tool to detect *S. stercoralis* in patients. This case may help clinicians to raise awareness of strongyloidiasis in non-endemic areas.

## Ethics Statement

The studies involving human participant was approved by the West China Hospital, Sichuan University. Written informed consent was obtained from the patient for the publication of any potentially identifiable images or data included in this article.

## Author Contributions

ZZ and JQ designed the study. JQ collected and interpreted the clinical data and drafted the manuscript. ZZ modified the manuscript and gave final approval of the version to be published. Both authors contributed to the article and approved the submitted version.

## Conflict of Interest

The authors declare that the research was conducted in the absence of any commercial or financial relationships that could be construed as a potential conflict of interest.

## Publisher’s Note

All claims expressed in this article are solely those of the authors and do not necessarily represent those of their affiliated organizations, or those of the publisher, the editors and the reviewers. Any product that may be evaluated in this article, or claim that may be made by its manufacturer, is not guaranteed or endorsed by the publisher.
